# IHE cross-enterprise document sharing for imaging: interoperability testing software

**DOI:** 10.1186/1751-0473-5-9

**Published:** 2010-09-21

**Authors:** Rita Noumeir, Bérubé Renaud

**Affiliations:** 1 Department of Electrical Engineering, École de Technologie Supérieure, University of Quebec, 1100 Notre-Dame West, Montreal, Quebec, H3C 1K3, Canada

## Abstract

**Background:**

With the deployments of Electronic Health Records (EHR), interoperability testing in healthcare is becoming crucial. EHR enables access to prior diagnostic information in order to assist in health decisions. It is a virtual system that results from the cooperation of several heterogeneous distributed systems. Interoperability between peers is therefore essential. Achieving interoperability requires various types of testing. Implementations need to be tested using software that simulates communication partners, and that provides test data and test plans.

**Results:**

In this paper we describe a software that is used to test systems that are involved in sharing medical images within the EHR. Our software is used as part of the Integrating the Healthcare Enterprise (IHE) testing process to test the Cross Enterprise Document Sharing for imaging (XDS-I) integration profile. We describe its architecture and functionalities; we also expose the challenges encountered and discuss the elected design solutions.

**Conclusions:**

EHR is being deployed in several countries. The EHR infrastructure will be continuously evolving to embrace advances in the information technology domain. Our software is built on a web framework to allow for an easy evolution with web technology. The testing software is publicly available; it can be used by system implementers to test their implementations. It can also be used by site integrators to verify and test the interoperability of systems, or by developers to understand specifications ambiguities, or to resolve implementations difficulties.

## Background

The Electronic Health Record (EHR) enables access to relevant diagnostic information in order to assist in health decisions; and this, independently from the geographic location of the point of access or the institution where the information was initially gathered. However, EHR is not a single system that can be provided by a single manufacturer. It is a virtual system that results from the cooperation of several heterogeneous distributed systems. Interoperability is therefore essential. Achieving interoperability requires the use of communication standards; it also requires common vocabularies, common semantics, as well as process flows that are agreed on. Therefore, ensuring interoperability requires various types of testing: testing peers' ability to communicate and exchange data; testing peers' ability to parse and extract information from the successfully exchanged messages; and testing peers' ability to react to the extracted information by changing information in their systems or by influencing subsequent workflow actions.

Interoperability challenges in healthcare are important: healthcare systems have to deal with extremely diverse clinical information such as diagnostic images, lab or cardiology results, as well as with various healthcare specific standards. Moreover, interoperability testing in healthcare is very new. To our knowledge, it started in 1999 with the first Integrating the Healthcare (IHE) connect-a-thon [1]. It is a face-to face testing event where hundreds of systems from various healthcare manufacturers test their software implementation of IHE profiles by executing real clinical scenarios. By putting in place this testing event, IHE has been a pioneer in healthcare testing.

In order to ensure interoperability and to conduct testing accordingly, detailed specifications are needed. One could think that medical standards such as the Digital Imaging and Communications in Medicine (DICOM) [2] and Health Level 7 (HL7) are enough to ensure interoperability. But medical interoperability history has demonstrated that this is not the case. These standards have existed for almost three decades without ensuring interoperability in healthcare. In fact, standards are necessary. Without them interoperability is simply impossible. However, standards alone are not sufficient. They specify how systems can exchange messages or information without specifying how messages can or should be combined to conduct a workflow. Also, to ensure their long term viability standards tend to encompass many if not all possible situations. Therefore, they suffer from various ambiguities and offer multiple possible choices that hinder interoperability.

Filling the gap between standards and systems implementation has required expensive, site-specific interface development to integrate even standards-compliant systems. To close that gap, a framework for the implementation of standards has become an urgent need that lead to the creation of the IHE initiative. IHE started in 1998 and was initially jointly sponsored by the Radiological Society of North America (RSNA) and the Healthcare Information and Management Systems Society (HIMSS). The first IHE integration profile, the Radiology Scheduled Workflow (SWF) provided a solution for integrating multiple systems involved in the fulfilment of a radiology order [3]. IHE follows an approach where care providers identify the key interoperability problems they face, and where healthcare manufacturers and information technology experts agree upon an implementation that uses established standards to provide a solution for each identified interoperability problem [1]. Interoperability demonstrations are regularly organised at major conferences worldwide, such as the annual meetings of RSNA, HIMSS and the American College of Cardiology (ACC). The main objective of such demonstrations is to inform and educate end users about existing integration profiles ready to solve their interoperability problems.

During the interoperability demonstrations, real clinical scenarios are executed by sharing health information between participating systems from various manufacturers. Therefore, to ensure the success of such demonstrations, participating systems test their interoperability capabilities in advance, during a weeklong live-testing event where participants' systems are gathered in the same place at the same time. During this face-to-face testing event, systems test their ability to exchange information with peer systems from multiple other vendors.

Again, to ensure the success of the live-testing event, participants test their implementation with a software-testing tool, beforehand. This testing tool consists of documents and software that simulates communication partners, in addition to providing test data and test plans. Succeeding in all tests for a specific actor in a specific integration profile is required to participate in the face-to-face testing event, and to subsequently participate in demonstrations.

Currently, IHE has expanded over several clinical domains, and its activities are solving, every year, new integration problems in domains such as radiology, laboratory services, cardiology, radiation oncology, ophthalmology and healthcare devices. Moreover, IHE is setting up the foundation for the EHR by enabling interoperability amongst care domains within a single healthcare enterprise and across many healthcare enterprises. For example, the Patient Identifier Cross-referencing Integration Profile (PIX) enables a system to query for other identifiers of a specific patient, and the Cross-Enterprise Document Sharing Integration Profile (XDS) addresses the needs for the registration, distribution and access across health enterprises of patient's clinical information. Several live-testing events are taken place around the world every year. Even though IHE testing early purpose was to support educational demonstrations, its uniqueness, its technical team experience and the large number of systems tested, have contributed to propel this event to the level of the de-facto testing in healthcare.

Amongst IHE integration profiles, XDS lays the architectural foundations for EHR. The XDS architecture enables patient's information, from separate care delivery systems, to be shared in the form of documents, between cooperating enterprises [4]. Another IHE integration profile, the Cross-Enterprise Document Sharing for Imaging (XDS-I) leverages the XDS architecture to enable the sharing of imaging information such as images and imaging diagnostic reports [5].

In this paper we discuss the interoperability concerns and describe the architecture of the XDS-I testing software. We first describe the XDS architecture and introduce how XDS-I enables the sharing of imaging information. Next, we briefly present the communication protocols and discuss the challenges they introduce. Then, we discuss the interoperability concerns, describe the software architecture and detail design decisions to overcome challenges and constraints. We also present the software functionalities and discuss their extensibility and limitations.

### XDS Architecture

The XDS architecture enables patient information to be shared, in the form of documents, between multiple institutions. It is document content neutral so that any type of document encoded in compliance with a widely accepted standard can be shared. Therefore, this document centric architecture offers mechanisms to publish query and retrieve specific clinical documents for a specific patient.

The architectural model is based on a central registry that holds metadata describing every published document. Documents reside in repositories and the registry metadata includes an address that allows a consumer system to retrieve a specific document from the repository where it is located. The central registry is responsible for storing information about documents. It maintains meta-data about each registered document and responds to queries about documents meeting specific criteria. The registry does not store the document itself. However, it maintains information about the location from which documents may be retrieved. Therefore, the architecture includes one or multiple distributed document repositories. A repository stores documents in a persistent manner and responds to document retrieval requests.

**Figure 1:**
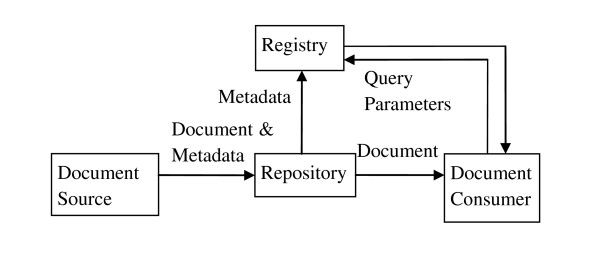
**XDS architecture and data flow**.

Systems that produce information relevant to patient's continuity of care, such as laboratory, cardiology, or radiology reporting systems, publish information as documents. Figure 1 depicts the basic architecture and data flow. Systems that are interested in accessing the patient's record query the registry for documents meeting certain criteria. Within the response to a query, the registry includes information about the document address, enabling the document consumer system to retrieve the document from its repository.

On the other hand, the XDS architecture allows systems to share information about the identity of the patient using a patient identity feed transaction. This transaction is not described here because we focus on the data flow for sharing images. The complete description of the XDS architecture can be found in the ITI-Technical Framework [3].

### Sharing of imaging information

Images represent important components of a patient's health record. Leveraging the XDS architecture to enable the sharing of imaging information allows the central registry and the document repository to treat imaging documents as any other clinical document. More importantly, a document consumer would be able to query and retrieve an imaging report as well as any another clinical document such as a laboratory result. Leveraging the XDS architecture requires specifying shared documents and metadata in addition to specifying additional process flow actions on some actors.

### Sharing of manifests

In order to publish a set of images, a manifest that contains references to those images is the document that is published. With this solution, the manifest is published and not the images. Medical images are encoded in conformance with the DICOM standard. Accordingly, images are grouped into Series that are grouped into Studies. Studies, Series and Instances of images are uniquely identified with Universal Identifier (UID). Therefore, the manifest is another type of DICOM object that contains the structure, in terms of UIDs, of the images that are referenced. Moreover, the manifest contains, at the Series level, the Application Entity (AE) of the DICOM server from which the Series can be retrieved. AE can also be considered as the name of that server.

### Process flow

**Figure 2:**
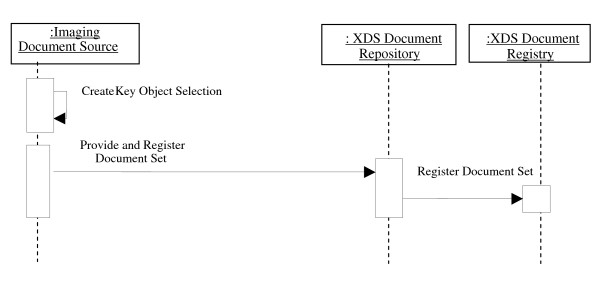
**Process flow for publishing a set of images**.

A system that wants to share a set of images would thus construct a manifest that references the images and submits that manifest to the repository/registry. Figure 2 shows the process flow followed by an imaging document source to share a set of images. Moreover, the publishing system, or the system whose AE title is referenced inside the manifest, is required to make the referenced images available to be retrieved.

On the other hand, a system that is interested in retrieving previously published images, starts by querying the registry and receives the address of the published manifest. Then, it retrieves the manifest and decodes it to get the list of referenced images. Each referenced object is specified by its study, series and instance UID along with the AE title where to retrieve it. The consumer can then issue a DICOM transaction, such as retrieve (C-MOVE) or a Web Access to DICOM Persistent Objects (WADO) request, to retrieve the images.

**Figure 3:**
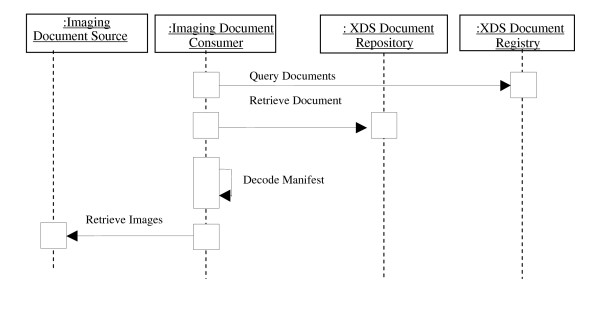
**Process flow for retrieving a set of images**.

A WADO request is an HTTP request to a WADO server requesting a specific DICOM instance using specific WADO query keys [2]. Query keys can be specified in a way to enable medical image streaming according to JPEG 2000 Interactive Protocol (JPIP) [6]. Figure 3 shows the process flow followed by an imaging document consumer to retrieve a set of images.

### XDS communication infrastructure

The XDS architecture is built on the Electronic Business Extensible Markup Language (ebXML) [7]. ebXML is a joint initiative by the Organization for Advancement of Structured Information Standards (OASIS) and the United Nations Centre for Trade Facilitation and Electronic Business (UN/CEFACT). Its goal is to create a global electronic market place where enterprises can find each other electronically, conduct business by exchanging XML messages in accordance to standard business process sequences and with clear business semantics. The ebXML specifications are intended to cover the entire business-to-business process.

A registry is a central piece of to the ebXML architecture. An ebXML registry provides a set of services that enables the sharing of information between trading partners. It maintains metadata for a registered item. Access to an ebXML registry is provided through standard interfaces exposed by Registry Services (RS). Moreover, ebXML Registry Information Model (RIM) defines the type of metadata that is stored in the Registry as well as the relationships among metadata classes. An ebXML registry serves as the index and a gateway for a repository to the outside world; it is a sort of a database for sharing relevant company information needed in ebXML business transactions, such as business process, order forms, or invoices.

The XDS architecture is built on the ebXML RIM and RS. This allows generic ebXML registry to be used as an XDS registry. However, XDS assigns the responsibilities of indexing a document and that of archiving it to separate actors: a Document Registry and Document Repository respectively. This separation of duties offers an important additional flexibility in the healthcare domain, which consists in having a single document registry indexing content for multiple document repositories. According to the XDS specifications, a document source submits the metadata and the documents to the document repository actor who forwards the metadata to the document registry actor. A detailed description about how ebXML has been specialized to build XDS can be found in [4].

Of relevance here is that XDS metadata specifications add constraints on the ebXML registry information model. While conforming to ebXML RIM, several XDS metadata content are derived from other medical standards data types such as HL7, and are encoded as such in the registry. Moreover, in addition to the definition of an XDS metadata vocabulary, additional requirements on metadata are defined such as required presence or uniqueness enforcement of specific data, as well as particular metadata content validation. Therefore, constraints are imposed on metadata and specific validation is required from the XDS registry.

Although XDS registry is needed to implement any XDS-I process flow, it is not specific to XDS-I. To the contrary, an XDS registry has many specific responsibilities, mainly with regards to metadata validation and query implementations, as defined by the XDS specifications. Therefore, it is common sense to reuse an XDS registry implementation in order to put in place the architecture needed for XDS-I testing. Such XDS registry implementation is provided by the National Institute of Standards and Technology (NIST) and is publicly available [8].

## Related Work

The objective of testing can be either to validate conformance to standards, or to validate interoperability according to integration profiles.

Conformance testing consists in validating that relevant requirements in a standard have been correctly implemented. It follows a black box approach where the tester sees the system under test as a closed box without having any knowledge about neither its internal structure nor its implementation. This is usually done by sending messages to the system under test and by observing its response in terms of acknowledge or response messages that are sent back. If at least one error is encountered, the system under test does not conform to the standard. However, the absence of errors, while a prerequisite to interoperability, does not guarantee it; this is the same thing as acknowledging that a standard is necessary to interoperability but not sufficient. Interoperability testing on the other hand is a way to ensure that different systems can co-operate to perform a specified business sub-process. By exchanging messages that conform to one or multiple standards, the co-operating systems react to the received information by changing their internal data or state, or by triggering actions or message exchanges with the same peer or others. Interoperability testing aims at observing that the system under test, not only can exchange information with peers, but also reacts correctly according to an agreed interoperability profile.

Conformance and interoperability testing are not new to the communication industry. One only needs to think of the Bluetooth technology for which many private companies offer interoperability testing. ebXML conformance and interoperability testing is another example from the electronic business industry. In this domain, interoperability is pushed a bit further: in order to support testing, the OASIS ebXML Implementation, Interoperability and Conformance Technical Committee has defined detailed specification for a test conformance framework [9] for which some implementations exist [10,11]. This test framework describes a standard architecture for the test system, as well as standard specifications for defining test scripts. Designing a new test case is eased and reduced to writing a new test script. But, interoperability testing in this case is defined as a way to verify that two implementations or more can interoperate according to an agreement which is compliant with the specification, along with additional restrictions. The main gap here is that the agreement is neither standardized, nor shared. It has to be defined every time heterogeneous systems decide to cooperate together. In the domain of robots communication, [12] describes a test suite for conformance and interoperability testing of web service communication between different robots. Although the test suite validates syntax, communication and message sequencing, the emphasis is put on reliable communication and therefore, the test suite provides tests for errors cases by simulating various forms of intentional communication errors.

In healthcare, conformance and interoperability testing are at their very early stages. As pointed out by [13], conformance testing, interoperability tools and techniques are needed in all healthcare domains to ensure the integration of healthcare enterprises. Also in [13], the authors reviewed many healthcare domains such as electronic health records and bio-imaging to analyse specific aspects where additional standards are needed in order to achieve automation; they pointed out that current standards deal only with syntactic issues whereas the disparate nature of healthcare vocabularies requires the development of semantic interoperability too. Very few projects exist for interoperability testing in healthcare. In [14], a test framework is proposed to design and execute testing of HL7 communication, document, and business layers. The business layer is described in terms of scenarios that are expressed in the HL7 specifications. These scenarios usually require the exchange of messages between two actors. Their test system can also act as proxy between two testing peers. This framework enables the fast and easy design of new tests. It follows the Upper-Lower Tester model described by [15] and tests only HL7 interoperability, as opposed to our system that is based on an actor approach [15] to test a specific interoperability profile using several communication standards for information exchange. The framework described in [14], as well as our system, propose tests for HL7 communication, document and business layers as opposed to document validation only for the Picasso platform [16]. This is a commercial tool that acts as a central interface between various communicating peers to transform a message from one version of HL7 to another. The Picasso interoperability platform is based on an internal HL7 version 3 structured representation of the data and uses style sheet transformation technology to transform data between different formats. In [16] a method for automatically validating such transformations is presented and discussed.

As mentioned earlier, two different approaches for testing are identified and discussed in [15]. The common approach to healthcare system testing is the Upper-Lower Tester. Using this approach, the system under test communicates with a Lower Tester via a specific communication protocol, and with the Upper Tester being the user or the business application under test. Although this approach is widely used, it suffers from many limitations: it usually allows the testing of one system and one message at a time. In other words, the testing environment does not simulate the working environment in which communication between multiple applications take place by exchanging a series of messages in order to collaboratively conduct a business process. Even when this approach is expanded to test a choreography, e.g. more than one message between more than one system as in [14], it still suffers from the lack of business level testing that can be achieved with the actor based testing. An actor is an application that has specific business responsibilities and communicates with other actors according to constrained messages that all cooperating actors agree on. Actor based testing allows the construction of an environment that simulates the real operation environment in which the system under test is expected to operate. Actor based testing does not suffer from the limitation of the Upper-Lower Tester. However, it requires the business process to be specified in terms of profiles between actors with identified responsibilities.

Our testing system is based on an actor testing approach. Testing can take place at the communication level and the content level by validating the content of exchanged messages. Moreover, testing can take place at a high business level by validating that the system under test has either changed its internal data or triggered data exchange with other parties. Validating the change to the internal data can be achieved by exchanging different messages with the tested system. Validating the triggering of data exchange can be achieved by simulating the other peers or by monitoring the communication with them. IHE testing is based on actor testing. The Mesa testing tools [17] have been used to test interoperability in preparation for connectathon [1]. IHE Gazelle project, also an actor based approach, is a multi-organization work in progress effort that allows the testing of multiple systems.

## Implementation

### Specifications

For defining the specifications of our testing system, we based our analysis on: 1- a detailed study of the XDS-I integration profile specific requirements in terms of additional communication constraints, semantic and business requirements; 2- the integration concerns as identified by a study [18] whose objective was to analyze the losses encountered in the U.S. automotive supply chain due to integration problems. Such losses, in the billions each year, are incurred due to: 1- the lack of information flows; 2- the flow of incomplete, inaccurate, or improperly represented data; 3- or the misinterpretation of received data [19]. In an attempt to analyze and study potential automations for this particular integration problem, a NIST report [18] has identified several integration concerns; these concerns are based on the Model for Open Distributed Processing (RM-ODP) [20]. This model is developed by the International Organization for Standardization (ISO) in collaboration with the International Electro-technical Commission (IEC) and the International Telecommunication Union (ITU). RM-ODP defines essential concepts necessary to specify open distributed processing systems from five viewpoints:

1-Technical: where integration aspects relate to the underlying communications, message structures and content as well as control flow.

2- Semantic: where integration aspects concern the consistent interpretation of the exchanged information which requires an agreement on common concepts and terms used to refer to those concepts.

3- Functional: where integration aspects concern behaviors of systems in consistency with their roles in the overall process; these concerns include the objects to be acted on and the actions to be done.

4- Policy: where integration aspects concern the ability to support the business process in an acceptable way; these aspects include security, reliability, availability, accuracy, and timeliness.

5- Logistical: where integration aspects relate to tradeoffs between limitations to integration and the overall value of the system such as cost, flexibility and openness.

Our system verifies technical, semantic and functional integration aspects. Likewise other IHE testing tools, the possibility of verifying not only technical aspects but also semantic and functional integration concerns is due to interoperability requirements that constrain communication standards and that are defined and detailed in integration profiles. These integration profiles are publicly available; they are also agreed on by almost all vendors in the targeted domains. Conversely, most other testing projects deal only with the verification of technical concerns. Even verifying the choreography, as specified by HL7 version 3, lies in our opinion under a technical concern as it can be considered a control conflict.

In Tables 1, 2 and 3, we describe the technical, semantic, and functional concerns respectively along with their impact on the specifications of our system. For each type of concerns a list of conflicts as identified in [18] are given in the first column; a brief definition of each conflict is provided in the second column along with examples from the healthcare domain for clarifications when necessary; in the third column we describe how testing for the specific conflict has influenced the specifications of our system.

**Table 1:** Technical concerns

Conflict name	Definition	Impact on specifications
Connection conflict	There is a disagreement at the communication level including the lower layers.	Since we do not execute conformance testing, we only require that the communication successfully takes place while verifying additional communication requirements when necessary. Such additional communication requirements include for example the presence of web addressing at the SOAP level as required by the integration profile.

Syntactic conflict	Different data structures or representations are used between peers.	In addition to verifying the structure of a message, we verify the presence of all required data, and the correct structure of any published document: published documents are constrained to be of specific types such as CDA or DICOM manifest.

Control conflict	The communication peers do not agree on their roles (e.g. which peer is the server) or in the flow of control of a communication interaction (e.g. immediate or deferred acknowledgment).	We do not perform any specific verification of this conflict, as we only require that the communication be successful.

Quality-of-service conflict	The behavior of a communication peer does not satisfy technical requirements derived from "policy" concerns, such as a timely response to a communication response.	Using a timeout, our system will consider the communication as failed, if not timely achieved.

Data consistency conflict	Peers do not consistently use information that is not directly communicated in the interaction (e.g. configuration data). In our case, such data include information about peers' addresses, procedure codes, document types and other codes that would generally be shared in a healthcare enterprise.	Our system requires that the tested system uses shared configuration information with the testing software.

**Table 2:** Semantic concerns

Conflict name	Definition	Impact on specifications
Conceptualization conflict	Communicating applications have incompatible representation of the same concept. Examples include how to describe an address, a person, a document.	IHE integration profiles define common concepts. In our system, concepts central to sharing documents are validated by NIST registry while those specific to sharing images are validated by our system. Amongst validated concepts is the manifest that must relate to specific images; this is validated by verifying the manifest content.

Conceptual scope conflict	An important concept is not communicated by one of the peers.	Important concepts are made required in IHE profiles and their presence is validated in communication transactions.

Interpretation conflict	The message has a different meaning to the listener than it does to the speaker; in other words, the technical communication is completely successful, but the intent is not fulfilled.	In IHE profiles, expected actions are specified and our testing software validates that expected actions have been accomplished mainly in two ways: if the receiving system is required to trigger a communication, the testing software awaits this and validates the communication content; if the receiving system is required to change its internal state, the testing software triggers a transaction to the tested system and validates the response content. When testing an imaging document source, the testing software verifies that the images referenced in a published manifest are available by issuing WADO transactions to the image archive. Likewise, when testing an imaging document consumer, the testing software awaits for image query from the tested system to ensure that the received manifest has been successfully decoded and interpreted.

Reference conflict	The communicating applications use different systems of reference for identical concepts. Examples include how to reference an imaging procedure, whether this is done with the accession number or the procedure id.	The testing software validates the structure used to reference images inside a manifest, as well as the consistent identification of the manifest i.e. the use of the manifest UID to identify the document inside the ebXML message.

**Table 3:** Functional concerns

Conflict name	Definition	Impact on specifications
Functional model conflict	Two applications have incompatible factorings of the process activity space: there may be a task that each expects the other to do ("nobody's job"), or a task that both expect to do themselves ("overlapping roles").	Roles and responsibilities are specified in the IHE integration profile. When testing a system, our software tests a specific role and therefore tests all responsibilities associated with that role as required by the profile.

Functional scope conflict	One party's behavioral model for a function contains more activities than the other party's model: 1- when the requester's model is larger than the performer's model, the performing application executes a subset of the expected behavior, leaving some expected tasks not done; 2- when the requester's model is smaller than the performer's model, the performing application executes unexpected activities as well as those requested. One example of functional scope from the IHE radiology technical framework relates to the Modality Performed Procedure Step (MPPS) transaction where the expected behavior of the receiving system is not specified in details; therefore this transaction is usually successfully received but does not trigger actions or state change as implicitly expected.	In the profile of interest here, functional scope conflict is avoided as far as the applications implement the required responsibilities of the specific role they play; these responsibilities are verified by the testing software as stated above.

Embedding conflict	The behavior of an application is affected by the integration with other peers. This happens when the application is capable of some adaptability in behavior which might be configured.	This kind of conflict is supposed to be detected and fixed by the testing engineers while using the testing software. The testing software does not detect en embedding conflict per se; but performing the testing with the help of our testing software simulates the integrated environment in which the application is supposed to operate in a real situation; therefore, an eventual embedding conflict would be fixed before deployment.

Intention conflict	The application is being used in a way its design did not anticipate, resulting in unexpected behaviors. This relates to differences in the details of the application specification versus the specification as needed for the role of that application in the larger system. This kind of conflict is hard to grasp. One example of such conflict is encountered in the way x-ray images are organized into series: one acquisition equipment may group multiple x-ray images into one series, while another one may put each image in a different series. Although both equipments have the right to do so, the receiving system may not be able to function with one or the other type of image grouping.	Even though the testing software helps in detecting some intention conflicts, such conflicts may arise anytime during the operation of the integrated larger system.

### Challenges: discussion and solution

XDS is built on ebXML and uses Simple Object Access Protocol (SOAP) [21] as its transport mechanism. ebXML as well as SOAP have evolved recently; this introduces challenges that will be discusses hereafter. On the other hand, NIST registry provides the validation and the functionalities of an XDS registry, but it imposes architectural constraints that will also be presented and discussed.

### Leveraging of NIST Registry

NIST Registry is accessible over the Internet; it will be used in our overall architecture to fulfil the role of an XDS registry because it provides an extensive validation for the registry functionalities, transactions and data.

However, in order to use NIST registry, any published document needs to belong to a patient that is already known to it; therefore, the patient identification that is used must be already known to NIST registry. As a solution, a patient is registered using a specific web page provided by the NIST registry, beforehand. The patient identification obtained is then used throughout testing sessions by including it in a specific configuration file.

### Different versions of the underlying communication protocol

XDS transactions are wrapped into SOAP message and transported over HTTP. However, SOAP has evolved recently to overcome some challenges. Its newer version includes the ability to attach binary parts in an optimized way; it also includes the ability to insert, part of the message and in a standard way, addressing information for more flexible networking topologies. This has a direct impact on our testing software.

In fact, the communication between the document source and the document repository involves the transmission of metadata along with documents whose original format may be in binary (i.e. DICOM manifest). With SOAP 1.1, two approaches are possible for handling the issue of binary data communication: 1- Embedding such data in XML as text-encoded (base64) octets which increases the message size and impacts performance; 2- referencing the binary data in the XML document, with the binary data bundled as an attachment (SOAP with attachments). The initial version of XDS adopted the second approach by specifying a standard way to bundle the ebXML message and the binary attachments in a MIME multipart package. This approach does not describe how intermediaries should deal with this referenced data, limiting therefore networking flexibility.

SOAP 1.2 brings a solution for these problems by adopting the Message Transmission Optimization Mechanism (MTOM) along with the XML-binary Optimized Packaging (XOP). XOP defines how to serialize the binary data as parts of a MIME multipart related packaging format, while MTOM describes how to serialize a SOAP envelope using XOP. More information on the dependencies of XDS on SOAP can be found in [4]. What is of relevance here is that two different versions of SOAP exist and each one is used in a different version of XDS. This variability introduces challenges because both versions need to be tested by the same software. We have designed the system with the ability to run the tests in either flavour.

### Different versions of specifications

With the new version of SOAP (version 1.2), and the release of a new version of ebXML (version 3.0), a new version of XDS (XDS-b as opposed to the initial version that was renamed XDS-a) has been designed to benefit from the advancements in these fields. From the testing point of view, this is a challenge. While, the process flow and medical content exchange is not changed, the underlying communication mechanism is different between the two versions. But, both versions need to be tested, even though the newer version is expected to deprecate the initial one. The definition of a new version of the specifications does not eliminate the need for testing of the version to be deprecated in the future. In fact, the initial version is being deployed presently in many national projects; therefore testing of systems cooperating according to this initial version will still be needed. The testing software is designed to test implementations according to one version or the other.

The initial version of XDS (XDS-a) is based on SOAP 1.1 and ebXML 2.1. It specifies the usage of SQL queries to query the registry. This specification requires the consumer system to know about the registry data model, therefore puts limitations on the evolution of that data model. In order to hide the registry data model from the outside world, stored queries have been introduced where the knowledge of the data model is no more needed at the consumer system. The end result is an infrastructure with two possible ways of implementing registry queries. Moreover, each query type has been designed with a different ebXML version: SQL queries are specified with ebXML 2.1 and stored queries are specified with ebXML 3.0. Therefore, XDS-a comes with 2 flavours of queries, each specified with a different version of ebXML.

The new version of XDS (XDS-b) is based on SOAP 1.2 and ebXML 3.0. ebXML data structure is different from the one of ebXML 1.2. Moreover, it uses Web Service Addressing specification (WS-Addressing) and provides informative Web Services Description Language (WSDL) contracts for its transactions [22]. WS-Addressing permits the specification of endpoint addresses as part of the SOAP header to allow routing flexibility. But most importantly, it enables an abstract separation between the application layer, the Web services messaging infrastructure layer, and the message transport layer. These abstraction layers allow the developer to concentrate on the application layer by using special framework such as Apache AXIS2 [23]. AXIS2 takes care of the infrastructure layer according to the rules set in the WSDL. Therefore, it simplifies the implementation and we have chosen to use it.

One last difference between the two versions is that the initial version uses HTTP GET to retrieve a document while the newer version uses Web Service Retrieve instead. The later uses MTOM and offers the possibility to retrieve multiple documents with a single request.

Our system design isolates the business logic from the communication mechanisms. The business logic consists in specific test flows along with specific validation, while high level transactions may differ by the underlying communication protocols. The software implements different versions of the same high level transactions and can instantiate a specific version depending on a user's setup.

### Web Services technology

XDS-a uses SOAP with attachments. SOAP, HTTP requests and HTTP retrieves can be implemented with standard Java API in addition to some external libraries. XDS-b brought in more advanced Web technologies that are not implemented in standard Java API. That is why we have decided that AXIS2 could bring an important value added.

AXIS2 is a core engine for Web services [23]. It provides the capability to add Web services interfaces to Web applications and can also be used as a standalone server application.

AXIS2 uses its own XML model called AXIOM (AXIS Object Model) for parsing XML. The SOAP implementation within AXIS2 is based on AXIOM. It is a pull parser as compared to DOM or XOM that are push parsers. This means that AXIOM controls the parser and builds the XML representation in memory only when specific information is needed as opposed to parsing the complete XML tree at once. Therefore, it can partially build the tree so unnecessary data won't be loaded in memory. This translates into a performance gain therefore, overcoming one of the major disadvantages of XML which is slow parsing.

We have decided to use AXIS2 because it takes care of the web infrastructure. As a result, we can focus on interoperability testing while mitigating the risk caused by the evolution impact of web technologies on our testing software.

### Architecture

The objective of our system is to test an application that can play the role of either an imaging document consumer or an imaging document source. Each of these two roles is tested separately and the tests are described in section 3. In order to implement the various tests, our system simulates the peers needed by the tested application. Moreover, it controls the data used during the tests, imposes a specific test flow and validates the content of all received transactions.

**Figure 4:**
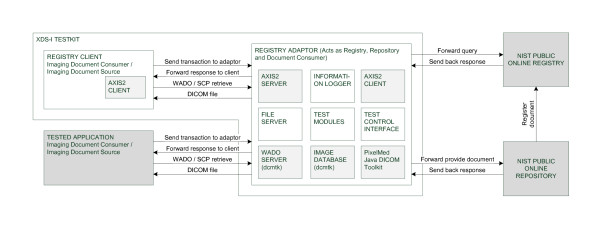
**Overview of the system architecture**.

An overview of the system architecture is depicted in Figure 4. Components with gray background are third-party software available for public use: DICOM Toolkit provides the functionalities of a DICOM archive [24]; pixelMed provides software tools for converting between multiple image and report formats [25]; AXIS2 provides a high level API for web services, SOAP and XML programming. Moreover, our system relies on NIST public registry to provide the functionality of an XDS registry. In the following sections, we describe the testing system's components.

### Registry adaptor

The adaptor is as a proxy to NIST registry/repository. It logs the SOAP messages and the attachments for testing purposes. It analyse the incoming SOAP messages to decide whether it is a submission request or a query. In case of a submission request, it archives the attached files in the local repository before sending the request to the registry. In this sense, it acts as a document repository as it archives the published documents.

It also receives requests for retrieving documents from a tested document consumer system. To serve a retrieve request, it forwards the query to NIST, modifies the response so it references the local documents, and sends the response to the query initiator.

When testing an imaging document source system, the adaptor acts as document consumer because it queries NIST for the submitted manifest.

### Simple Publisher

This software component constructs a manifest along with a submission request to be sent to the registry. It retrieves images from the WADO server, creates the manifest, and publishes it to the registry.

It is used by the testing software for publishing the manifest to NIST Registry in order to test an imaging document consumer system. It can also be used by a software developer to publish his/her own manifest to the registry.

### Registry Client

This registry client is a utility software that can be used by a software developer to interact with NIST Registry. It can be used to publish or to query for a document.

### Image Database/DICOM Retrieve SCP

This is a DICOM Server provided by a third party software [12]. It accesses and manages a database for archiving DICOM instances.

### WADO server

The WADO Server receives a HTTP GET request and returns the required DICOM instance. The WADO server contacts the DICOM SCP Server to retrieve the required DICOM instance. It converts the instance before sending the response according to the requested content type. For its conversion needs, it uses the DICOM Toolkit [12] for converting reports into XML or HTML and uses PixelMed [13] for converting images into JPEG.

### Information logger

The logger logs information gathered at various steps of a test so it can be verified at the end of the test execution. Information logged includes all messages content (requests as well as responses), third party console output (such as DCMTK output), and explicit test output. Logger also saves attachments.

### Web Test Control Interface

The control interface provides the GUI to the user and controls the test flow. It presents to the user available tests and instantiate a specific test execution that is associated with the chosen test. During a test execution, it presents to the user information on expected next actions and the test state. A test is divided into multiple stages. This controller calls 'next Stage' or 'cancel' method on the test. Upon reaching the final stage, an evaluation is performed and the evaluation result is presented to the user. The test instance logs valuable information in a XML format; also, it evaluates the XML logs using an XSLT.

### Test modules

These modules group different classes and software responsible for specific test flows and test evaluation.

### AXIS2 Client

AXIS2 Client uses AXIS2 API for sending SOAP messages to a remote WEB Server.

### AXIS2 Server

AXIS2 Server uses AXIS2 to act as a WEB service. It provides to AXIS2 a file describing its WEB Server endpoints allowing the received message to be redirected to the appropriate class capable of handling the business logic.

### Design for dealing with two different versions of specifications

The main differences between the two different versions are the following:

Profile A: SQL Query, ebXML 2.1, SOAP 1.1, SOAP with attachment, HTTP GET, and Stored Query that requires ebXML 3.0.

Profile B: Stored Query only, ebXML 3.0, SOAP 1.2, MTOM/XOP, WS-Addressing, and WS-Retrieve.

**Figure 5:**
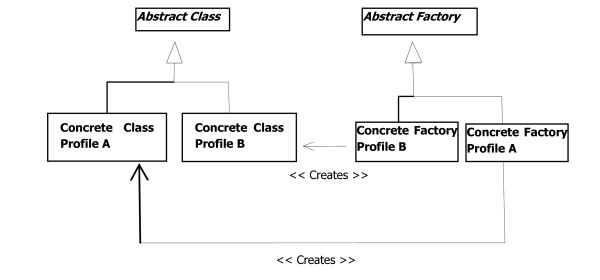
**Overview of the software design for dealing with various versions of standards**.

Our design goal is to have a single version of the common business logic for an easier maintenance and evolution. Therefore, this logic has been factored out in abstract classes whereas specific logic has been implemented with sub-classes. The Abstract Factory [26] design pattern has been used as shown in figure 5 to manage the instantiation of the family of classes. In fact, there are two different versions of factories; each is responsible of instantiating classes for a specific version of the profile. Both factories inherit from the same abstract factory. Furthermore, factory instantiation is managed by a singleton that reads information from a configuration file and decides the type of factory to instantiate. This design decision ensures that, both client and web service, function according to the same version even though they run each in a completely different environment. A mechanism to override the default ebXML version to be used has also been implemented to allow for various underlying communication versions.

## Results

### Tests description

#### Testing an imaging document consumer actor

To test a specific actor, the testing software simulates the other actors involved in the test flow. For testing an imaging document consumer actor, the software simulates the document registry, the document repository, and provides an imaging document source actor. This test consists of the following steps:

##### Preparation of the testing data

The testing software builds its internal DICOM archive by loading in it the image instances to be published. It ensures that its archive is able to receive connections. The user is asked to use a software tool, part of the testing software package, in order to publish a manifest referencing the images in the archive.

##### Query to the registry

The imaging document consumer system under test is required to query the registry. It sends a query to the XDS adaptor that forwards it to NIST registry after validation; the XDS adaptor receives NIST response, changes the reference to the published document so it points to the local one and sends it to the system under test.

##### Retrieve of the manifest

The imaging document consumer system under test is required to extract, from the response to its query, the reference to the manifest of interest and to retrieve it. The testing software logs every retrieved manifest for evaluation.

##### Image retrieve

The testing software starts its internal archive so it can accept retrieve transactions (by C-MOVE or WADO). The imaging document consumer system under test is required to parse the manifest, to extract the referenced UIDs and to use them in order to retrieve, at least one of the referenced DICOM instances, using either WADO or DICOM C-MOVE from the testing software archive. The output of the archive software (DICOM Toolkit) is logged in a file for evaluation.

#### Testing an imaging document source actor

**Figure 6:**
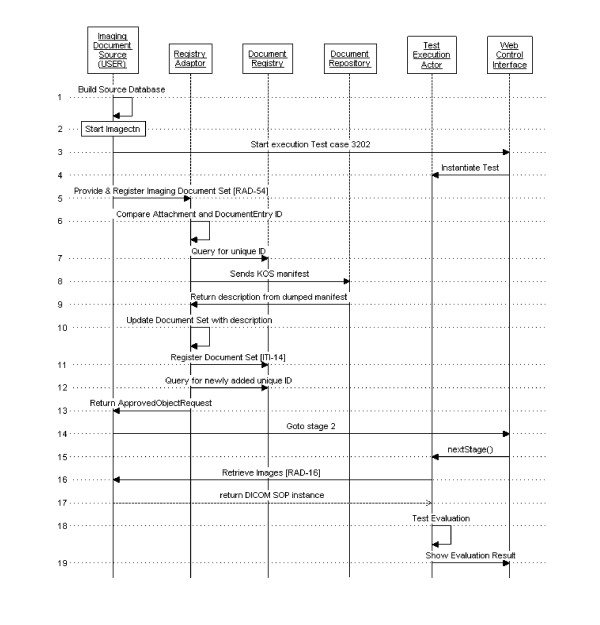
**Process flow for testing an Imaging Document Source**.

The process flow of this test is depicted in Figure 6, where references to IHE transactions are indicated within square brackets. The test's steps are as follows:

##### Preparation of the testing data

The imaging document source system under test is required to send a submission request with a DICOM manifest to the XDS adaptor. The manifest references images that are managed by the system under test.

##### Publishing of the manifest

The testing software verifies that the newly submitted manifest has not been previously submitted to NIST registry. It does so by querying the NIST registry for the identification of the manifest to be published. After the registration with NIST registry is completed, the testing software verifies that the registration step with NIST registry was successfully completed by querying for the new document identification.

##### Image retrieve

The testing software parses the received manifest, extracts all referenced UIDs and retrieves from the imaging document source system under test, all DICOM instances that are referenced in the published manifest. The test completes successfully if at least one instance is retrieved. The image retrieve transaction is done using either DICOM C-MOVE or WADO. Two tests are provided to support each flavours of this transaction.

## Discussion

Building the testing software on top of AXIS2 is a first step towards a better and full support of WEB Service technologies as they become more common. In the future, mechanisms that enable the testing software to make use of WSDL would be implemented. At the moment this article is written, neither NIST registry nor AXIS2 infrastructure were ready to fully use WSDL. However, as WSDL technology can be expected to be in place in the very near future, the testing software would largely benefit from relaying on it.

The design of the testing software has been carried out to reduce the impact of schema modifications and to allow easy extension either for adding new tests or adding new transactions. Schemas are retrieved directly from the WEB and used for validating messages' content. This ensures that the latest version of the schema is used. On the other hand, the implementation of the testing software can be easily extended to include new tests or to include new transactions. Adding a new test has been simplified by the design of the software; the test execution has been factored in a single class that can be extended. Adding a new transaction requires the specialization of some message-type classes along with the implementation of their adequate evaluation.

## Conclusions

We have presented the architecture of a web application for testing interoperability in healthcare. The proposed software provides functionalities to test peers involved in sharing images between different institutions. We have also presented various challenges encountered and discussed the elected solutions. Furthermore, we have described the web technologies underlying the XDS framework, corner stone of the Electronic Health Record that is in deployment in several countries.

This software, available from [27,28], can be used by system implementers to test their implementations. It can also be used by site integrators to verify and test the interoperability of systems, peers in the same healthcare process. Moreover, developers can use it to understand specifications ambiguities, if present, and to resolve implementations difficulties.

The software is built on evolving technologies. XDS specification is evolving; it evolved recently to benefit from new versions of ebXML and of SOAP; XDS specifications also evolved to enhance interoperability, maintainability, flexibility and efficiency: one such example was the introduction to Stored Queries as compared to ad-hoc Queries. XDS underlying technology is also evolving; web technologies are continuously developing and they directly impact XDS and XDS-I. The architecture and design of the testing software were worked out to mitigate the impact of such changes. More precisely, relying on Apache AXIS2 infrastructure to effectively handle web communications, message packaging and XML efficient parsing, is expected to have contributed to isolate the software from the web infrastructure layer. Thus, we think that the proposed testing software is ready to support new web development easily, and that it will evolve without major difficulties.

## Availability and requirements

Project name: IHE-XDS-Imaging

Project home page: http://sourceforge.net/projects/ihe-xds-imaging/

Operating system(s): Platform independent

Programming language: Java

Other requirements: Java 1.6, Apache Tomcat 6.0, dcmtk 3.5.4

License: GNU GPL version 3

Any restrictions to use by non-academics: None

## Competing interests

The authors declare that they have no competing interests.

## Authors' contributions

RB implemented the refactoring of the software to integrate AXIS2 and to support two versions of XDS. RN carried out the test specifications, the analysis and interpretation of data as well as writing the article. All authors read and approved the final manuscript

## Authors' information

RN has been actively involved with Integrating the Healthcare Enterprise, at the international level, since its inception. She is the author of several IHE Integration profiles in radiology. In particular, she authored the Cross-enterprise Document Sharing for Imaging. She also directed the implementation of IHE testing software with the support of Canada Health Infoway. The testing software includes XDS-I testing tools, Patient Identification Cross-Reference (PIX) using Health Level 7 (HL7) version 3 (v3) and Patient Demographics Query (PDQ) with HL7 v3. She also directed the XDS-I demonstration during the Radiological Society of North America (RSNA) scientific assembly and annual meeting in November 2006 along with the face-to-face interoperability testing event (connect-a-thon) in preparation to the demonstration.

RN is a professor at the Department of Electrical Engineering of the University of Quebec, École de Technologie Supérieure in Montreal. She holds a Master's and Ph.D. degrees in Biomedical Engineering from École Polytechnique, University of Montreal. Her main research interest is the Healthcare Information Technology, specifically, Interoperability, Electronic Patient Record, Security, Information Confidentiality and Image Processing. Dr Noumeir has provided consulting services in architecture analysis, workflow analysis, technology assessment and image processing for several software and medical companies including Canada Health Infoway.

RB graduated recently in Electrical Engineering with a major in computer science from University of Quebec, École de Technologie Supérieure in Montreal. During a research internship under the supervision of Dr Noumeir, he participated to the development of the IHE XDS-I testing software by re-engineering the software in order to integrate web services and to support multiple versions of the integration profile. He also provided technical support for developers and implementers that used the testing software.
